# Movement pattern definitions for resistance training behavior measurement in diabetes

**DOI:** 10.3389/fcdhc.2024.1447595

**Published:** 2024-08-27

**Authors:** Elise C. Brown, Lon J. Kilgore, Kyle Pierce, Allan Knox, Joshua L. Haworth

**Affiliations:** ^1^ Department of Public and Environmental Wellness, Oakland University, Rochester, MI, United States; ^2^ Department of Curriculum Development & Delivery, Kilgore Academy, Halfway, MO, United States; ^3^ Kinesiology & Health Science, Louisiana State University Shreveport, Shreveport, LA, United States; ^4^ Department of Exercise Science, California Lutheran University, Thousand Oaks, CA, United States; ^5^ Department of Human Movement Science, Oakland University, Rochester, MI, United States

**Keywords:** diabetes mellitus, type 2, resistance training, health communication, activities of daily living, measurement, public health, health education

## Abstract

Type 2 diabetes can result in debilitating vascular complications, and resistance training (RT) is an effective therapy for improving cardiovascular outcomes. However, only 10–30% of adults meet the public health guidance for RT. While current RT guidelines focus on targeting major muscle groups, guidance specific to simplified movement categorization may augment understanding of RT programming and improve uptake and outcomes. Current movement pattern definitions and descriptions lack clarity, accuracy, and consistency. This paper proposes movement definitions and descriptions to be used for RT intervention design and prescription, and includes the following categories: hip, knee, ankle, vertebral column, vertical push, horizontal push, vertical pull, and horizontal pull. These categories are intended to aid clinicians, researchers, and trainers in RT surveillance and RT intervention design for improving vascular complications in type 2 diabetes. Simplified RT program design using these categories may also facilitate greater RT program understanding and adherence for patients.

## Introduction

Type 2 diabetes (T2D) is a metabolic condition of impaired insulin uptake and sometimes production (1). From 1980 to 2014, diabetes prevalence increased four-fold from 108 to 422 million adults, and the majority of cases were T2D (2). Increased prevalence is concerning as micro- and macrovascular complications accompany this condition, including retinopathy, neuropathy, and nephropathy (3). Common macrovascular complications include cardiovascular, cerebrovascular, and peripheral vascular disease.

Chronic resistance training (RT) reduces vascular comorbidities and cardiometabolic risk factors in T2D (4–6), and some benefits manifest after a single session (4). Despite these benefits, only 10–30% of adults in the USA, Scotland, Australia, and Finland meet RT public health guidance (7–10). RT is “a category of exercises intended to increase muscular strength; most commonly the applied resistance takes the form of a barbell, dumbbell, weight machine, or simple gravity (*bodyweight exercise*)” ( (11), p. 166). This form of exercise drives unique structural and metabolic adaptations, including increased muscular strength, power, muscle cross-sectional area, muscle activation, neural drive (12), bone mineral density, basal metabolic rate (13), and anabolic hormone secretion (14).

RT additionally improves endothelial function, hypertension, glycosylated hemoglobin concentrations, blood lipid levels, and body fat % in prediabetes and T2D (4, 15, 16), although these effects vary by race, ethnicity (17), sex (18), and age (19). RT intervention characteristics such as frequency, intensity, and use of single- or multi-joint exercises also influence intervention effectiveness (15). RT protocols often lack key component descriptions such as rest periods, movement speed, implements utilized (*e.g*., barbell or weight machine) (20–22), and included exercises (23, 24). This heterogeneity across studies makes replication and dosage determination difficult. In order to optimize health benefits for people with T2D, accurate and consistent RT definitions are warranted.

When selecting exercises to include in interventions, a focus on physical function rather than muscle groupings may be beneficial. Qadir et al. (15) found that most RT interventions that reduced cardiometabolic risk in this population primarily incorporated multi-joint exercises. Previous studies using combinations of multi- and single-joint RT improved endothelial function (16, 25), cardiac autonomic function, and inflammation in T2D (25). Furthermore, multi-joint exercises mimic natural movements performed in everyday life better than single-joint isolation exercises.

Activities of Daily Living (ADL) are often listed in exemplar form: moving a table, loading a washing machine, toilet use, and self-hygiene (26). While these activities are understandable in terms of outcome, in a functional context they involve pushing, pulling, bending, and twisting movements. Some research suggests that diabetes impairs real-world physical function by reducing capacity to perform ADL (27). Conversely, RT replicating ADL movement patterns may improve ADL competency (28, 29).

Research is currently underway to test modification of the Muscle-Strengthening Exercise Questionnaire using movement patterns instead of muscle groupings (data not published) (30). Thus, the present paper will explore publicly used nomenclature and proposes movement definitions and descriptions intended to enable uniform understanding and consistent application for RT interventions to reduce cardiovascular comorbidities in T2D.

## Issues in RT exercise categories

Training muscles in isolation, using single-joint exercises, is a prominent characteristic of bodybuilding programs as competitors are assessed for muscularity, definition, size, shape, proportion, symmetry, and balance of whole-body development. In the general population, such training is generally performed intending to alter physical appearance by increasing muscle hypertrophy (31). Isolation training appears prominently in public health guidelines. The 2018 Physical Activity Guidelines for Americans (32) recommended performing muscle-strengthening activities (*i.e*., RT) working all major muscle groups at least twice a week (32). When major muscle groups are defined as the legs, hips, back, abdomen, chest, shoulders, and arms, such presentation may be interpreted as a RT isolation exercise approach. Most of the public can likely identify generic body segments, but the public’s understanding of anatomy is limited (33–35). Additionally, it is unclear if the public can identify exercises specific to body parts or muscle groups. Alarmingly, the vast majority of U.S. physical education teacher education programs do not offer RT principles and methods coursework (36). A similar circumstance exists in medical education (37–40). If two primary sources of health and fitness information are unfamiliar with RT fundamentals, any expectation of the public understanding and applying RT principles and methods to their lives may be unfounded.

It is generally assumed that physical education or sports science graduates will have mastered fitness delivery methods. However, in a study examining the knowledge of RT principles and methods in high school physical education teachers and coaches, neither group demonstrated minimum competencies to design and implement safe RT programs (41). More recently it was reported that less than 18% of teachers took courses in resistance training techniques, weight room safety, and resistance training programming during their Physical Education Teacher Education (42). These findings indicate a fundamental knowledge gap in the primary professionals who are tasked with educating the public on physical literacy. Furthermore, low levels of anatomical knowledge in the public is of concern (33–35), as an inability to identify anatomical structures (*e.g.*, muscles and bones) may hamper the ability of the public to understand RT concepts. Classifying and describing resistance exercises objectively and uniformly may enable more effective conveyance of information from fitness and health professionals to the public.

**Figure 1:**
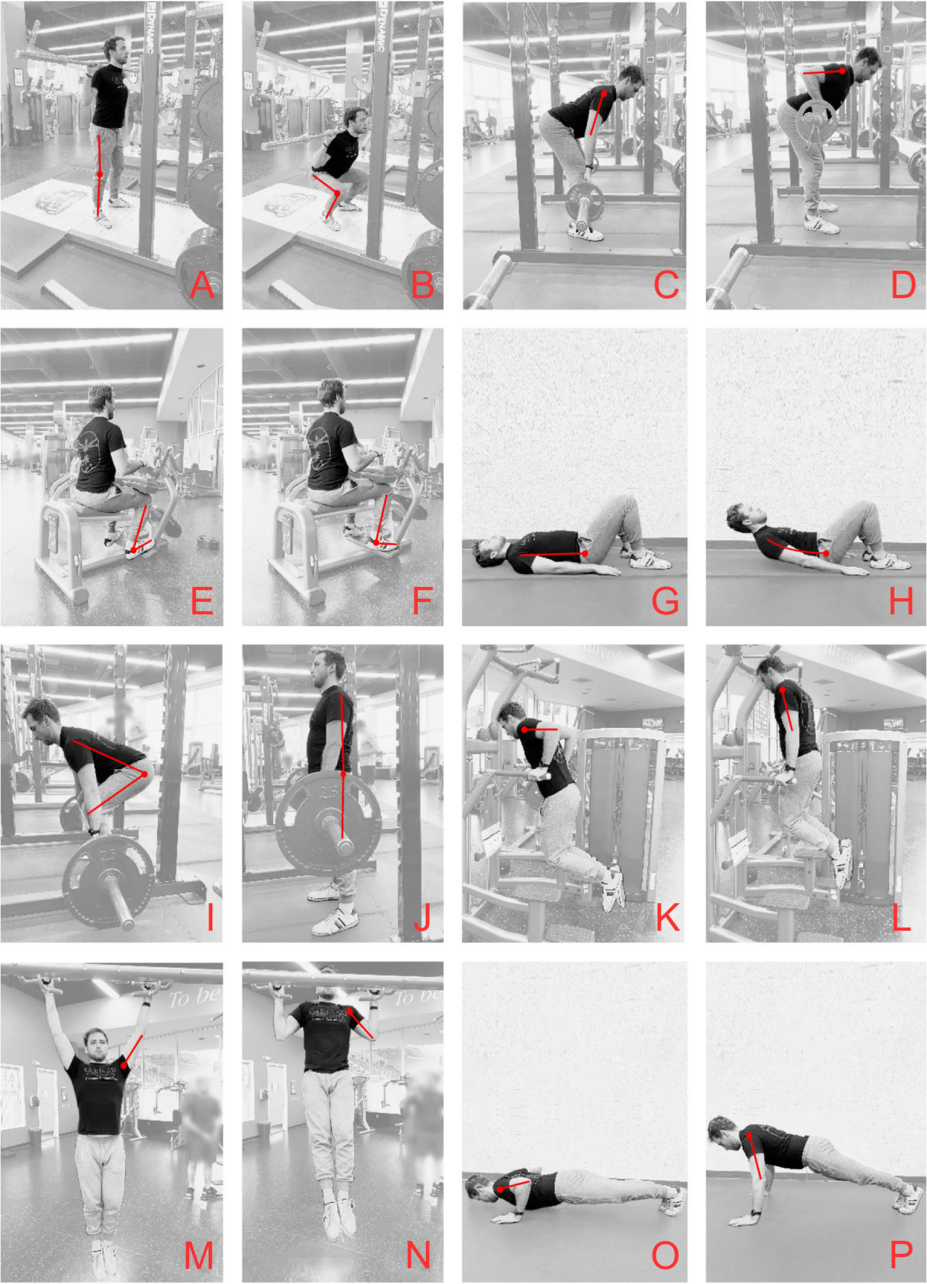
Exercise examples for each movement category; **(A)** Back Squat start, **(B)** Back Squat finish, **(C)** Bent Row start, **(D)** Bent Row finish, **(E)** Calf Raise start, **(F)** Calf Raise finish, **(G)** Crunch start, **(H)** Crunch finish, **(I)** Deadlift start, **(J)** Deadlift finish, **(K)** Dip start, **(L)** Dip finish, **(M)** Pull Up start, **(N)** Pull Up finish, **(O)** Push Up start, **(P)** Push Up finish. For categoric assignment see **Table 1**.

An example of an exercise often misclassified in effect is the Bent Row (**Figure 1 2A, B**). Here, the hands grasp a barbell, while the hips flex forward until the vertebral column is at or slightly above parallel to the floor. Once the “bent” position is achieved, the body remains isometrically contracted, and the barbell is pulled up from the floor toward the vertebral column until the bar contacts it at about the level of the xiphoid process of the sternum. It is common to see this exercise described as only affecting the latissimus dorsi, trapezius, and posterior deltoids, as only the arms moving at the shoulder are considered (43). This approach omits the contribution of elbow flexors and ignores the roles of other muscles in providing isometric postural stability and movement control. A system of simple movement categorizations accessible to a general audience may improve RT uptake and outcomes.

Movement occurs when muscles provide force at moveable joints, and basic motor patterns are learned in childhood from formal and informal physical experiences (44). Movement pattern categories currently in use are variable, but generally describe the application of force around the primary joint most moved during an exercise. “Pushing” moves segments away from the center of gravity of the body, while “pulling” moves segments toward the center of gravity (45). Although a push or pull can be generated by any muscled joint of the body, description and categorization of these terms can be confusing. When considering pushing or pulling involving the arms, pushing is generally characterized by elbow extension, while pulling is characterized by elbow flexion. This simplicity becomes inaccurate if used with compound movements.

Over the past two decades, several non-scientists have proposed movement pattern categories for RT exercises (46–48). Push-pull sequencing of exercises is common in RT programs created for athletes (49–51), military personnel (48), and first responders (48, 52, 53). Despite its popularity, objective definitions for these commonly used terms are sparse in the literature (54, 55). Castanheira et al. (55) defined the term, push-pull, as exercises that work non-synergist muscles in the upper body. In 2003, Boyle (47) proposed seven categories for sports performance exercises (1): hinge hip-dominant (2), knee-dominant (3), rotational (4), horizontal push (5), horizontal pull (6), vertical push, and (7) vertical pull movements. In 2018, Mullins (46) proposed three additional categories specific to general fitness exercise movements as follows: stability, gait, and cross-pollination (*i.e.*, combination of movement patterns). Lastly, in 2021, Tumminello (48) proposed eight exercise movement categories for the training of military personnel and first responders (1): jumping and landing (2), throwing and striking (3), locomotion (4), rotation (5), pushing (6), pulling (7), knee bending, and (8) hip hinging. In a clinical setting, Rosenberg et al. (54) categorized movement patterns as (1) push (2), pull (3), hinge (4), squat, and (5) core. All of these proposed categorizations suffer from inconsistency, weak and inaccurate descriptors, gaps in applicability, and lack of consensus.

Commonly, patients with T2D are referred to fitness facilities that are equipped primarily with RT machines (56). RT machines focus on primary movers and minimize recruitment of motor and isometric stabilizing musculature (43). A simple and logical system of categorization and description that is useful for trainers, and understandable by patients, may improve RT prescription quality, uptake, and outcomes.

## Movement patterns for public health

Some RT exercises, such as isolation machine exercises, fit into categories intuitively. Other exercises, such as compound exercises, need careful consideration in terms of direction of body or object movement(s), movement planes, and joint(s) moved by active musculature. Every RT movement requires recruitment of specific muscle groups (57), and this specificity makes categorization difficult to create and apply universally as exercises may fit into multiple movement categories in current systems.

Practical movement patterns for RT proposed here include Vertical Push (VPush), Horizontal Push (HPush), Vertical Pull (VPull), and Horizontal Pull (HPull). These patterns occur at the ankle, knee, hip, vertebral column, shoulders, and wrists. Choosing the joint and muscles most dominant in the target exercise (*i.e.*, primary movers) as categorical components, rather than identifying every minor synergist, motor stabilizer, and isometric stabilizer active at every joint, creates a less informationally-dense system that may be easier for patients to understand. Exercise examples for these terms along with primary movement patterns, joints, and muscles involved are presented in **Table 1**. Images of exemplar exercises are presented in **Figure 1**.

**Table 1:** Categorical organization of example exercises.

Fig. #	Exercise Name	Equipment	Movement Type	Direction	Primary Joint	Secondary Joint	Tertiary Joint	Primary Movers
1	Back Squat	Barbell	Push	Vertical	Knee	Hip	Ankle	*Knee* - rectus femoris, vastus intermedius, vastus lateralis, and vastus medialis; *Hip* - adductor magnus, biceps femoris, gluteus maximus, gluteus medius, semimembranosus, semitendinosus; *Ankle* - gastrocnemius, soleus, tibialis posterior, flexor digitorum longus, flexor hallicus longus, fibularis
2	Bent Row	Barbell	Pull	Horizontal	Shoulder	Elbow		*Shoulder* - deltoid; *Elbow* - biceps brachii, brachialis, brachoradialis
3	Calf Raise (seated)	Machine	Push	Vertical	Ankle			*Ankle* - gastrocnemius, soleus, tibialis posterior, flexor digitorum longus, flexor hallicus longus, fibularis
4	Crunch (abdominal curl-up)	Bodyweight	Pull	Horizontal	Vertebral Column			*Vertebral column* - rectus abdominis, external oblique, internal oblique
5	Deadlift	Barbell	Pull	Vertical	Hip	Knee		*Hip* - adductor magnus, biceps femoris, gluteus maximus, gluteus medius, semimembranosus, semitendinosus; *Knee* - rectus femoris, vastus intermedius, vastus lateralis, and vastus medialis
6	Dip	Bodyweight	Push	Vertical	Shoulder	Elbow		*Shoulder* - coracobrachialis, deltoid, pectoralis major; *Elbow* - anconeus, trieps brachii
7	Pull Up	Bodyweight	Pull	Vertical	Shoulder	Elbow		*Shoulder* - deltoid, latissimus dorsi, supraspinatus; *Elbow* - biceps brachii, brachialis, brachoradialis
8	Push Up	Bodyweight	Push	Horizontal	Shoulder	Elbow		*Shoulder* - deltoid, pectoralis major, pectoralis minor, coracobrachialis; *Elbow* - anconeus, triceps brachii

See **Figure 1** for exercise movements.

Hip movements refers to *lower body actions directly involving muscles moving the hip joint.* The Deadlift (*i.e*., free weight) and Back Extension (*i.e.*, weight machine) are exercises included in this category. Both exercises are used under the premise that they recruit the gluteals and hamstrings. The machine exercise fits this idea well. However, the Deadlift, while meeting hip movement criteria, carries additional accessory functions. There are a few centimeters of knee extension in the movement along with recruitment of motor and isometric stabilizers (*i.e*., hip adductors, abductors, and flexors). The Deadlift also invokes isometric vertebral muscle contraction during hip extension and this further affects the posterior muscles attached to the glenohumeral joint and shoulder girdle (58). Thus, while Deadlift prime movers place it in this category, Deadlifts are often included in training for their knee or vertebral column developing capacity. Exercises within this category are single-joint (*e.g.*, Back extension) but may include multi-joint exercises (*e.g*., Deadlift) with primary movers functioning at the hip.

Knee movements refers to *lower body actions directly involving muscles moving the knee joint.* The Leg Extension and Squat both involve knee extension, and this is commonly considered the primary joint involved and musculature developed. The Leg Extension fits this concept well, but the Squat involves multiple joints, the hip very profoundly, and the ankle to a lesser degree (59). Isometric contraction of the vertebral musculature adds motor stability and control. Exercises within this category are single-joint (*e.g.*, Leg Extension) and multi-joint (*e.g.*, Squat) with primary movers also at the hip.

Ankle movements refers to *lower body actions directly involving muscles moving the ankle joint.* Calf Raises are an example of an ankle movement. Ankle movements included in common exercises are plantar- and dorsiflexion. Eversion and inversion are more common in rehabilitative settings (60). While often assumed that exercises in this category will be single joint movements, in every exercise where the patient is standing and there is movement at the knee and hips, ankle muscles are also involved as synergists or stabilizers (61).

Vertebral column movements refers to *upper body actions directly involving muscles that move the torso.* Exercises in this category are restricted to those acting to move the vertebral column without direct hip and shoulder involvement, the most familiar of which may be the Crunch. Many exercises that are stated to target muscles of the torso may also involve movements of the hips. Such is the case for the Leg Raise, which involves lying supine, flexing the hip, and moving the extended legs upward. The only movement that occurs here is hip flexion via the hip flexors. However, it also requires the isometric contractions of the rectus abdominus to stabilize the torso (62). In such cases, as movement only occurs at the hip, it should be classified as a hip movement.

Vertical Push (VPush) movements are *actions applying force to an object that subsequently moves away from the body in an upward or downward direction, relative to the body’s center of gravity and postural alignment.* Vertical pushing can occur at almost any joint, and exercises in this category include the Dip (VPush - shoulder), Calf Raise (VPush - ankle), and Back Squat (VPush - knee, hip, and ankle).

Horizontal Push (HPush) movements are *actions applying force to an object that subsequently moves away from the body in a forward or rearward direction relative to the body’s center of gravity and postural alignment*. Horizontal pushing can occur at almost any joint, and exercises in this category include the Bench Press (HPush - shoulder and elbow), Leg Press (HPush - hip, knee, and elbow), and Push Up (HPush - shoulder and elbow).

Vertical Pull (VPull) movements are *actions applying force to an object that subsequently moves in an upward or downward direction toward the body’s center of gravity and postural alignment*. Vertical pulling can occur at almost any joint and exercises in this category include the Pull Up (VPull - shoulder and elbow), Upright Row (VPull - shoulder and elbow), and Deadlift (VPull - the hip, knee, and ankle).

Horizontal Pull (HPull) movements are *actions applying force to an object which subsequently moves toward the body in a forward or rearward direction relative to the body’s center of gravity and postural alignment.* Horizontal pulling occurs at almost any joint and exercises in this category include the Bent Row (HPull - shoulder and elbow) and Leg Curl (HPull - knee).

## Discussion

This paper proposes an organization of RT movements for use in public health, specifically for reducing vascular comorbidities in T2D. To ensure that all major muscle groups are trained, public health messaging could include ‘do exercises that push and pull each major joint at least twice a week, with most exercises involving more than one joint.’ Human movement largely occurs in a multi-joint manner. An organized approach to description and categorization can ensure that machine isolation exercises included comprehensively provide whole-body training. However, multi-joint RT exercises that mimic these movements are more likely to improve physical function, increase muscle activation and strength, and, consequently, reduce metabolic stress and injury risk compared to single-joint exercises (63, 64). The descriptors presented here provide a structure ensuring programming balance across body segments and muscle groups. Further, the descriptors may enhance understanding of which exercises to work what body part and for what purpose. This is especially important as previous research shows that a stronger understanding of exercise correlates to improved compliance and adherence (65).

Classifying resistance exercises by movement pattern rather than muscle group may help patients better conceptualize exercise technique and programmatic outcomes compared to naming muscle groups. If an RT program balances pushing and pulling movements, this approach should aid in correcting muscle imbalances and reduce injury risk (66, 67).

Movement pattern classification may improve training status assessment, critical for RT prescription (68). Commonly, training status is assessed by self-report of uninterrupted time that the trainee has been performing RT in years and months. Technique and strength level are additional methods of assessing training status (68, 69). Testing one upper body push, one upper body pull, one squat variation, and one hip hinge movement have been recommended, and categories proposed in the current paper may be preferential (68).

Movement pattern categorization may simplify program design. Movement pattern categorization may facilitate improvement of a specific ADL or understanding of other exercises similar to ADL. Patients/practitioners can use categories to select exercises within the same category to add to the training stimulus, choose replacement exercises per individual needs, or strength can be appropriately balanced in functional outcome.

Populations with T2D require considerations of their pathological status when making exercise selections. For example, when foot ulcers are present, resulting from peripheral neuropathy, non-weight bearing exercise is recommended (70). Use of devices such as a forefoot offloading shoe may complicate standing exercise selection further (71). In these situations, seated machine weights exercises provide less aggravation of lesions, as pressure applied to the feet is not required.

Despite the benefits of movement categorization, limitations exist. First, any system of categorization of movements faces challenges when considering exercises that clearly involve more than one movement pattern and joint. An example is the Snatch (*i.e.*, weightlifting competition lift) includes a modified Deadlift (VPull, hip 1°, knee 2°, ankle 3°), Shrug (VPull, shoulder 1°), and Overhead Squat (VPush, knee 1°, hip 2°, ankle 3°). Categorizing resistance exercises into individual joint movement patterns for health surveillance purposes and exercise prescription may effective with machine-based RT, as the complex nature of many free-weight exercises requires considered placement of some exercises into multiple categories. Another challenge may be variations of an exercise it designated category. For example, the barbell moves vertically in the Bench Press, yet the supine body position on the bench leads to the movement categorized as a horizontal push away from the anterior surface of the torso (HPush, shoulder 1°, elbow 2°). This categorization applies to low positive and negative angles of the Incline and Decline Bench Presses, however, more vertical equipment settings create close resemblances to a VPush. It is tempting to suggest that above a 45° angle setting be considered vertical and less than 45°considered horizontal, but more consideration is needed. Lastly, the forces applied across body weight, free weights, resistance bands, machines, and cables vary substantially, and may change the exercise categorization (72).

When selecting exercises for a RT program and communicating them to patients, using movement types, direction of movement, and utilized joints rather than muscle groups may simplify the programming process and improve patient understanding and adherence. The system presented here may result in improvements in strength, leading to enhanced ADL function, and better vascular health in those suffering from T2D by making RT seem less intimidating.

## Data Availability

The original contributions presented in the study are included in the article/supplementary material. Further inquiries can be directed to the corresponding author.
